# Regulatory Roles for Long ncRNA and mRNA

**DOI:** 10.3390/cancers5020462

**Published:** 2013-04-26

**Authors:** Armen R. Karapetyan, Coen Buiting, Renske A. Kuiper, Marcel W. Coolen

**Affiliations:** Department of Human Genetics, Nijmegen Centre for Molecular Life Sciences (NCMLS), Radboud University Nijmegen Medical Centre, P.O. Box 9101, Nijmegen 6500 HB, The Netherlands; E-Mails: A.Karapetyan@gen.umcn.nl (A.R.K.); C.Buiting@gen.umcn.nl (C.B.); RA.Kuiper@gen.umcn.nl (R.A.K.)

**Keywords:** non-coding RNA, epigenetic, mRNA, polycomb, remodeling, multifunctional, regulatory, transcript, lncRNA, lincRNA

## Abstract

Recent advances in high-throughput sequencing technology have identified the transcription of a much larger portion of the genome than previously anticipated. Especially in the context of cancer it has become clear that aberrant transcription of both protein-coding and long non-coding RNAs (lncRNAs) are frequent events. The current dogma of RNA function describes mRNA to be responsible for the synthesis of proteins, whereas non-coding RNA can have regulatory or epigenetic functions. However, this distinction between protein coding and regulatory ability of transcripts may not be that strict. Here, we review the increasing body of evidence for the existence of multifunctional RNAs that have both protein-coding and *trans*-regulatory roles. Moreover, we demonstrate that coding transcripts bind to components of the Polycomb Repressor Complex 2 (PRC2) with similar affinities as non-coding transcripts, revealing potential epigenetic regulation by mRNAs. We hypothesize that studies on the regulatory ability of disease-associated mRNAs will form an important new field of research.

## 1. Introduction

RNA molecules are best known for their ability to convey genetic information encoded in the DNA into the synthesis of specific proteins. This messenger function makes RNA an essential player in today’s DNA/RNA/protein world. It is commonly believed that our current DNA/RNA/protein world was preceded by a so-called *RNA-world*, a term first used by Gilbert in 1986 [1]. This world was based primarily on RNA molecules, which stored genetic information similar to DNA, and catalyzed chemical reactions similar to enzyme proteins in today’s world [2,3]. The *RNA-world* hypothesis has implicated a crucial role for RNA in the origin of life. Also in today’s DNA-based life, the function of RNA molecules is not limited to being a messenger for protein synthesis. In fact, only about 1–2% of the RNA present within a human cell is protein-coding, the remainder being non-coding RNA (ncRNA). The vast majority of this ncRNA is ribosomal RNA (rRNA) and transfer RNA (tRNA)—both involved in the process of translation [4]—as well as mitochondrial RNA (mtRNA) transcribed from DNA present in the mitochondria. In addition, and especially thanks to recent advances in massive parallel sequencing, the near entire repertoire of RNA molecules has now been identified. Important work by the ENCODE Consortium on the characterization of the complete RNA profile of human cells has shown that about 62% of genomic bases is represented in RNA molecules [5]. To date, this has resulted in the annotation of 13,249 unique long non-coding RNAs (lncRNAs) *versus* the 20,447 known protein-coding loci (GENCODE v15) with lncRNA numbers likely to increase further in later releases of GENCODE [6]. From an ever-increasing number of functional studies it has become apparent that lncRNAs—transcripts over 200 nucleotides in size—are involved in the regulation of gene expression at many levels, ranging from changing the epigenetic state of genes to influencing mRNA stability and translation. Also in the context of cancer, many lncRNAs have been shown to possess tumor suppressive or oncogenic properties [7,8,9,10,11,12,13,14,15,16,17]. This implies there is a much more complex role for RNA in cancer than previously anticipated. This review highlights both the differences and similarities between protein-coding and long non-coding transcripts. The roles of short RNA molecules (such as miRNAs) and their involvement in cancer are excellently reviewed elsewhere (e.g., [18,19,20,21,22]). Importantly, we summarize evidence for multifunctional roles for protein-coding transcripts. These multifunctional roles warrant a further (re-)investigation of deregulated transcripts in cancer, at the protein level and at the regulatory level.

## 2. Non-Coding *versus* Coding RNA

For most mRNAs ample evidence for their protein coding ability exists. Likewise, an ever-growing list of publications proves the involvement of lncRNAs in diverse aspects of gene regulation. Despite this major discrepancy in function, lncRNAs are in many ways very similar to mRNAs. The majority of active lncRNA genes are occupied by the same histone modifications as protein-coding genes, are synthesized by the same RNA polymerase II transcriptional machinery, 5' capped and are often spliced with similar exon/intron lengths [23,24]. Moreover, most long non-coding transcripts are polyadenylated [25,26,27]. Alternatively, some lncRNAs are generated via alternative pathways, and are for example not polyadenylated and likely to be expressed by RNA polymerase III [25,28], or excised during splicing [29]. Still, most known lncRNAs and their biogenesis pathways are indistinguishable from mRNAs. Global analyses of long non-coding transcripts did reveal a general bias towards a two-exon structure and localization in the chromatin and nucleus [30]. They are also expressed at lower levels and more frequently in a cell type specific manner compared to mRNAs [31]. Still, there is a significant overlap between transcript expression levels and distribution of coding and non-coding RNA. Only, their lack of protein coding ability and conservation is differentiating lncRNAs from mRNAs [26,32]. These are therefore the main criteria from telling both types of transcripts apart.

**Figure 1:**
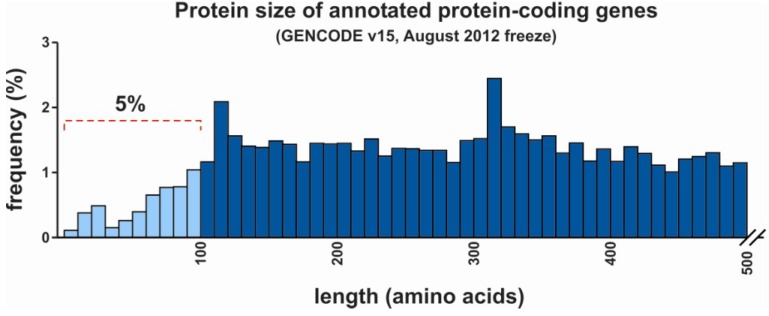
Size distribution of human proteins. Out of all annotated proteins derived from the protein-coding gene list in the GENCODE database (version 15, August 2012 freeze, GRCh37—Ensembl 70), five percent are less than 100 amino acids in size (1,039 out of 20,640). In this analysis, only the largest protein size was included when multiple isoforms were listed for a single gene ID.

*Protein-coding ability—*Proof of protein-coding ability can be obtained from experiments such as Western blotting using specific antibodies or via mass spectrometry. For example, in 2012, about one-third of all annotated human protein-coding genes were supported by peptide hits derived from mass spectrometry spectra submitted to PeptideAtlas [6]. This still leaves a large gap of evidence for many supposedly translated mRNAs. In contrast, finding proof of the inability of non-coding RNA to be translated into proteins is much harder. Bánfai and colleagues have shown that many annotated lncRNAs that are expressed at levels similar to mRNAs indeed lack mass spectrometry evidence, but still some did reveal peptides indicating they may be wrongly annotated as non-coding [33]. Theoretically, each open reading frame (ORF) containing a start and stop codon can give rise to a polypeptide or protein. To discriminate protein-coding from non-coding transcripts a minimum length of the ORF is generally being used. For example, the FANTOM consortium that analyzed the mouse transcriptome described coding RNA to have an ORF of at least 300 nucleotides (nt; *i.e.*, 100 amino acids) [34]. Similarly, the human transcriptome was analyzed by another consortium called H-Invitational that used a cutoff of 60 nt (20 amino acids) [35]. Unfortunately, these arbitrary cutoffs are far from ideal and have resulted in numerous incorrectly annotated RNAs for several reasons. Firstly, ncRNAs are likely to have an ORF by chance [36]. For example, a group of well documented lncRNAs including *H19*, *Xist*, *Mirg*, *Gtl2*, and *Kcnq1ot1* all contain ORFs longer than 100 codons, while they do not code for protein [37]. Secondly, transcripts with an experimentally proven ability to encode for proteins shorter than 100 amino acids, will be falsely considered as non-coding. Many of such known short proteins are involved in critical pathways in immunity, cell signaling and metabolism [38]. In fact, about five percent of all currently annotated proteins are less than 100 amino acids in size, which would all be incorrectly annotated using this cutoff (Figure 1). Lowering the threshold below 100 amino acids would allow the inclusion of very small known human proteins such as sarcolipin (SLN) [39] or ribosomal protein L41 (RPL41) with protein sizes of 31 and 25 amino acids, respectively [40]. Noncanonical, yet functional ORFs down to 11 amino acids have now been reported, indicating the possible existence of a new class of mRNAs [41]. However, setting the border of the ORF at a very low number of amino acids would obviously misclassify many ncRNA as coding RNA.

*Sequence conservation—*Instead of measuring the length of the ORF one could also examine the evolutionary conservation of the ORF. If the ORF of a novel transcript shows homology with other known proteins this indicates that the RNA could function as mRNA, while novel, non-conserved ORFs are likely to occur by chance and often do not function as protein-coding [42]. However, more recent research has revealed a frequent lack of conservation in newly identified protein-coding exons [43]. A further complicating factor is the common evolution of protein-coding genes, or copies thereof, into ncRNAs, such as pseudogenes. For example, the *Xist* gene evolved from a protein*-*coding gene and therefore still shows great overlap with mRNA features and a strong conservation [44]. Other pseudogenes have even been shown to be resurrected into protein-coding genes, further complicating the feature discrimination between mRNAs and lncRNAs [45].

*LncRNAs versus untranslated regions of mRNAs—*Interestingly, a recent study revealed significant similarities between lncRNAs and the 3' untranslated regions (3' UTRs) in protein-coding RNAs in structural features and sequence composition [46]. Both lncRNAs and 3' UTRs obviously lack protein-coding capacity and are intron-poor. Importantly, also the secondary structure predictions were highly similar between lncRNAs and the 3' UTRs of protein-coding transcripts, most likely due to a similar (lower) GC content. Also thermodynamically, lncRNAs were more similar to UTRs than to coding sequences [47]. Moreover, direct sequence comparisons revealed highly similar hexamer compositions in lncRNAs and 3' UTRs, which differed significantly from 5' UTRs or ORFs [46]. 

In conclusion, although lncRNAs and mRNAs do differ in their protein-coding ability, the above-mentioned facts about lncRNAs reveal a high degree of similarity between lncRNAs and mRNAs, or parts thereof. LncRNAs have been shown to play critical regulatory roles in diverse cellular processes including chromatin remodeling, transcription, post-transcriptional processing, as well as intracellular trafficking [48,49,50]. The presence of the intriguing parallels between the lncRNAs and mRNA raises the question whether protein coding transcripts may be able to fulfill regulatory functions similar to lncRNAs.

## 3. Regulatory Functions of lncRNAs and mRNAs

LncRNAs appear to be involved in nearly all aspects of gene regulation, including X-inactivation, imprinting, epigenetic regulation, nuclear and cytoplasmic trafficking, transcription, mRNA splicing and translation [51]. Through these involvements, lncRNAs have shown to be important players in a wide range of biological processes, such as proliferation, cell cycle, apoptosis, differentiation and maintenance of pluripotency [52]. Participation of lncRNAs into this wide range of processes can be explained by the ability of transcripts to fold into stable secondary structures, which in many cases dictate their functions [51]. Based on known examples, several functions have been proposed for lncRNAs. At the simplest level, lncRNAs can serve as decoys, preventing the access of transcription factors and other proteins to the chromatin [53,54]. In a scaffold model, lncRNAs can bring together multiple protein partners to form ribonucleoprotein complexes. Importantly, the concept of RNA as molecular scaffold is likely to be a more common mode of action as hundreds of lncRNAs have been identified to form ribonucleic protein interactions with multiple protein partners [15,55,56,57]. Finally, lncRNAs can function as guides for the proper localization of specific regulatory protein complexes in *cis* (on neighboring genes) or in *trans* (distantly located genes). The protein complexes brought on by the lncRNAs can act as epigenetic repressors and activators, as well as transcription factors [58].

Knowledge on how lncRNAs search for selective sites in the genome and how they interact with chromatin or target RNAs is slowly accumulating. LncRNAs can interact with RNA molecules via the formation of complementary hybrids [8,59,60]. They can also directly bind DNA by forming stable triplex structures via base-pairing [53,61] or by displacing one of the DNA strands and forming so-called R loops [62]. Alternatively, sequence-specific DNA-binding proteins can guide lncRNAs to target regions in the genome [63]. Recently, a novel mechanism of lncRNA targeting via chromosomal looping has been described for *HOTTIP* lncRNA [64].

For more detailed information about the mechanisms of lncRNAs action we refer to excellent reviews by others [65,66,67,68]. Also, their involvement in gene deregulation in cancer has been thoroughly reviewed elsewhere [9,10,69]. However, such regulatory roles are not solely attributed to non-coding transcripts. Also protein-coding transcripts have been shown to be involved in a number of regulatory mechanisms. Of course, many examples of *cis*-regulatory functions of mRNAs are known—mostly residing in the non-coding regulatory elements (untranslated regions, or UTRs)—and involve the regulation of stability, splicing and translation of the transcript [70,71,72]. Regulatory elements in the 5' UTR can play an important role in the control of translation initiation. Length, GC-content and secondary structures all affect translation efficiency [73,74]. Likewise, the 3' UTR can contain elements that are important in transcript cleavage, stability, translation and mRNA localization. The 3' UTR serves as a binding site for numerous regulatory proteins as well as miRNAs [75,76,77,78].

Importantly, mRNAs can also affect other genes or gene products via *trans*-regulatory functions. Below, we describe known and putative *trans*-regulatory functions of mRNAs and compare them to known lncRNAs with similar functions. Each example is also mentioned in Table 1.

**Table 1:** Regulatory functions of lncRNAs and mRNA and their type of interactions.

Function	Interaction				lncRNA^	mRNA	Mechanism	References
	RNA	miRNA	protein	unknown				
**structural**			•		*SATIII*		forms nuclear stress bodies by attracting splicing and transcription factors to *SATIII* repeats	[79]
			•		*NEAT1*		forms paraspeckles as large foci directly after transcription	[80]
			•			*H2B*	forms HLBs and Cajal bodies	[81]
			•			*VegT*	integral part of cytoskeleton at vegetal side in *X. laevis* oocytes	[82]
**transcriptional control**			•		*MEG3*		enhances p53 binding to promoters	[83]
			•		*MALAT1*		interacts with splicing factors to influence the localization and action	[84]
			•		*GAS5*		decoy for the glucocorticoid receptor	[54]
			•		*DHFR-minor*		prevents DHFR transcription via triple helix formation and TFIIB interaction	[53]
			•			*SRA*	co-activator for many nuclear receptors and transcription factors	[85,86,87,88,89,90,91,92,93,94]
**transcription elongation**			•		*7SK*		binds and inhibits P-TEFb, thereby blocking RNAPII elongation	[95,96,97,98,99]
			•			*HIC*	binds and activates P-TEFb by displacing 7SK RNA from inhibitory complex, allowing RNAPII elongation	[100]
**miRNA sponge**		•			*PTEN-P1*		binds miRNAs that also target PTEN, thereby increasing PTEN protein levels	[101]
		•			*HULC*		binds amongst others miR-372, thereby increasing PRKACB protein levels	[102]
		•				*VCAN*	binds miR-133a, miR-199a*, miR-144 and miR-431, thereby increasing protein levels of CD34 and FN1	[103]
		•				*CD44*	binds miR-328, miR-512-3p, miR-491 and miR671, thereby increasing protein levels of COL1α1 and FN1	[104]
**RNA degradation**	•				*1/2sbsRNAs*		imperfect base-pairing with Alu elements in UTRs of mRNA, thereby attraction STAU1 and initializing STAU1-mediated decay	[60]
	•					speculative	imperfect base-pairing between Alu elements in two mRNAs, thereby attraction STAU1 and initializing STAU1-mediated decay	[105]
**translational control**	•		•		*lincRNA-p21*		imperfect base-pairing with mRNA can directly impair translation and/or can attract translation inhibitors	[8]
	•				*PU.1-antisense*		processed RNA binds sense *PU.1* transcript and stalls translation	[106]
	•					*BCMA-AS*	blocks translation of the sense *BCMA* transcript	[107]
			•		*BC1*		interacts with eIF4A and PABP and blocks their interaction, thereby repressing the general translation machinery	[108]
			•			cytoskeletal mRNAs	inhibit translation by interaction with the RNA-binding domain of PKR, resulting in PKR phosphorylation events	[109]
			•			*P23/TCTP*	inhibit translation by interaction with the RNA-binding domain of PKR, resulting in PKR phosphorylation events	[110]
			•			*VEGFA, TPM1, IFN-γ, TNF-α*	UTR interacts with PKR, thereby inhibiting translation	[111,112,113,114]
			•			*p53*	interacts with MDM2, thereby preventing p53 degradation and promoting *p53* translation	[115]
**unknown **				•	*PCAT1*		*trans*-regulates many genes, including *BRCA2*	[116]
				•		*PHB*	3' UTR has unknown *trans*-regulatory role	[117]
				•		*RNR*	3' UTR has unknown *trans*-regulatory role	[118]
				•		*c-myc P0*	5' UTR has unknown *trans*-regulatory role	[119]
**guide for epigenetic** **enzymes**			•		*HOTTIP*		interacts with WDR5/MLL complex	[64]
			•		*HOTAIR*		interacts with PRC2 and LSD1-CoREST complex	[55]
			•		*ANRIL*		interacts with PRC1 and PRC2 complexes	[15,57]
					*HOTAIRM1*		interacts with PRC1 and PRC2 complexes	[120]
			•		*KCNQ1OT1*		interacts with PRC2 complexes and G9a	[56]
			•		*AIR*		interacts with G9a	[121]
			•		*pRNA*		recruits DNMT3b to rDNA promoters	[61]
			•			many ^§^	many mRNAs interact with PRC2 complex components	^§^

^: listed lncRNAs serve as examples for each functional group; ^§^: Reanalysis of our data [unpublished], Guil *et al.* data [29] and Zhao *et al*. data [122] revealed many mRNAs, see also Section 4.

### 3.1. Structural Function

LncRNAs can serve as structural scaffolds involved in the formation of nuclear domains. The first described non-coding RNA with a structural role is *Satellite III* (*SATIII*) [79]. *SATIII* is involved in the formation of nuclear stress bodies (nSBs) when cells are subjected to thermal, hypertonic or chemical stresses [123]. These cellular stresses change the heterochromatin state of *SATIII* repeats on chromosome 9q11‑12 to a euchromatin state. After transcription, *SATIII* RNA remains within the locus and recruits serine-arginine rich splicing factor SF2/ASF and several heat shock transcription factors like HSF1 and SAF-B to form nSBs [124]. *SATIII* was even shown to be sufficient for the formation of nSBs in the absence of a stress trigger [81]. A second lncRNA with an architectural role within the nucleus is nuclear-enriched autosomal transcript (*NEAT1*). *NEAT1* is a 3.7 kb long unspliced, polyadenylated transcript that is localized at the edges of SC35 domains in paraspeckles, which are found in all cells in interphase [125,126]. *NEAT1* was concluded to be essential for the assembly, maintenance and structural integrity of these paraspeckles [80,126,127].

Not only ncRNAs, but also mRNAs have been shown to perform architectural roles for cellular substructures. Two of these nuclear structures are the histone locus bodies (HLBs) and the associated Cajal bodies. The HLBs are known to harbor large amounts of histone pre-mRNA and histone 3'-end processing components [128], whereas the Cajal bodies contain small nuclear ribonucleoproteins (snRNPs) and are suggested to generate and recycle these proteins [129,130]. The *de novo* formation of both these nuclear components was shown to be induced by *histone 2b* (*H2B*) pre-mRNA [81]. In the same paper, spliced RNA Polymerase II transcripts are suggested to contribute to the morphogenesis of splicing speckles by functioning as a scaffold for pre-mRNA splicing factors. Another good example of an mRNA with a structural role is *VegT*, found in *Xenopus laevis* oocytes [131]. The *VegT* transcript was shown to be an integral part of the cytokeratin cytoskeleton at the vegetal cortex of the oocytes and responsible for the localization of *Vg1*, *Bicaudal-C* and *Wnt11* mRNAs at this position. Depletion of *VegT* mRNA therefore resulted in the delocalization of these mRNAs [131]. Furthermore, the acquired disruption in the cytokeratin cytoskeleton network could be rescued by injecting exogenous *VegT* mRNA [82].

### 3.2. Transcriptional Control

A second level of lncRNA-directed regulation is by *(co-)transcriptional* control. Here, the recruitment of RNA polymerase II, transcription factors and/or co-factors to gene promoters is facilitated or prevented by long non-coding RNAs. The lncRNA *MEG3* activates the *p53* tumor suppressor gene and the *growth differentiation factor 15* (*GDF15*) gene by enhancing p53 binding to the *GDF15* gene promoter, thereby inhibiting cell proliferation [83]. While *MEG3* is expressed in many normal human tissues, reduced levels of *MEG3* are frequently observed in a variety of cancers and associated with hyper-proliferation [14,132,133]. Another example is the abundant lncRNA *MALAT1*, which is frequently upregulated in many cancers and can regulate alternative splicing by modulating the phosphorylation of serine/arginine-rich splicing factors (SRSFs) [12,134,135,136]. Depletion of *MALAT1* altered the localization and activity of these splicing factors, leading to altered splicing patterns for a set of pre-mRNAs [84]. The lncRNA *GAS5* contains a hairpin sequence motif, mimicking a DNA binding site of the glucocorticoid receptor, thereby serving as a decoy to release the receptor from the DNA and preventing transcription of metabolic genes [54]. In case of the human *dihydrofolate reductase* (*DHFR*) gene, a lncRNA initiated from the upstream *DHFR-minor* promoter inhibits the assembly of the pre-initiation complex at the major promoter by forming a stable triple helix complex with promoter sequences, as well as through direct interactions with the general transcription factor IIB (TFIIB) resulting in the silencing of the *DHFR* gene [53,66].

The human *Steroid Receptor RNA Activator* (*SRA*) transcript was initially identified as a ncRNA that co-activates the Progesterone Receptor [86]. More recently, *SRA* RNA has been confirmed to co-activate many nuclear receptors, including estrogen (α and β), androgen, glucocorticoid, retinoic acid (α), peroxisome proliferator activated receptors (δ and γ), thyroid and vitamin D receptors [87,88,89,137], reviewed in [85]. Additionally, it was shown that *SRA* RNA can enhance the activity of transcription factors like MyoD and GATA3 [90,91]. It is thought that *SRA* ncRNA functions as a scaffold for nucleoprotein complexes with both positive regulators (e.g., receptor co-activator SRC-1, RNA helicases p68 and p72, pseudo-uridine synthases Pus1p and Pus3p [86,88,90,92,93,138]) and negative regulators (such as the SMRT/HDAC1 Associated Repressor Protein SHARP or the SRA stem-loop interacting RNA-binding protein SLIRP [89,94,139]). With the discovery of three new isoforms of *SRA* it was shown that these could also be translated into the protein SRAP [140]. Considering the fact that these longer *SRA* isoforms include the same core sequence as needed for the regulatory RNA function, this RNA was concluded to be bi-functional. Deregulated *SRA* RNA levels have been implicated in a variety of cancers [141,142,143,144,145,146]. Interestingly, high expression levels of the SRAP protein were shown to be a predictor for positive outcome in breast cancer [147]. 

### 3.3. Transcription Elongation

Transcriptional pausing is a well-known phenomenon, where RNA polymerase II (RNAPII) becomes trapped downstream of the transcriptional start site (TSS) and is unable to escape into productive elongation [148]. P-TEFb, the positive transcription elongation factor, plays an essential role in facilitating RNAPII escape from this paused state. When recruited to promoters, P-TEFb phosphorylates the *C*-terminal domain (CTD) of RNAPII, allowing the escape into productive elongation [148]. *In vivo*, P-TEFb is present in two states: an active P-TEFb form, associated with Brd4 and other factors, and in an inactive ribonucleoprotein from, referred to as 7SK snRNP, containing a 331-nt non-coding RNA known as *7SK* snRNA. RNase footprinting and mutagenesis experiments have indicated that *7SK* contains a high degree of secondary structure, with stem-loops at both the 5' and 3' ends [96,148,149,150]. The 5' stem loop binds P-TEFb as well as the Hexim1 protein, which acts to inhibit the kinase activity, while the 3' stem-loop binds the Larp7/PIP7S protein, which, in addition to a methylphosphate capping enzyme (Mepce), stabilizes the RNA [95,96,97,98,99,151,152]. For a long time the mechanism of P-TEFb release from the inhibitory complex was not known. However, a recent study has demonstrated the important role of *HIC* mRNA for P-TEFb activation [100]. The 3' UTR of *HIC* mRNA binds to and activates P-TEFb by displacing *7SK* RNA from inhibitory complex. Analysis of the secondary structure of *HIC* mRNA 3' terminal region revealed the existence of hairpins resembling similar structures within *7SK* RNA [100]. It is speculated that other mRNAs with similar secondary structure may exert the same function and multiple P-TEFb containing RNPs exist [100]. 

### 3.4. miRNA Sponge

MicroRNAs—a large class of small ncRNAs—have emerged as a critical element in gene regulation by interacting with incompletely complementary sequences in target messenger RNAs [66,153,154]. They function by annealing to complementary sites on the coding sequences or 3' UTRs of target gene transcripts, where they promote the recruitment of protein complexes that impair translation and/or decrease the stability of mRNA, ultimately leading to a decreased target protein abundance [153,154]. Aberrant expression of miRNAs has been linked to many cancer types as well as other human diseases [155,156]. There is now evidence that the inverse mechanism may also take place, whereby mRNA levels can affect the distribution of miRNAs. Such RNA molecules can compete for miRNA binding, thereby acting as a miRNA sponge or decoy independent of a possible protein-coding function (reviewed in [157]). Natural miRNA sponges were first discovered in plants [158] and more recently also in virally infected primate cells [159], and in human cells [101]. The miRNA sponge/decoy function has been recently described for a number of lncRNAs. Specifically, the 3' region of the *PTEN-P1* lncRNA was found to bind the same set of regulatory miRNA sequences that normally target the tumor-suppressor gene *PTEN*, alleviating the *PTEN* mRNA repression and allowing its translation into the tumor-suppressor protein PTEN [66,101]. Another interesting example is lncRNA *HULC* which may act as an endogenous miRNA sponge that down-regulates a series of miRNAs, including miR-372. Inhibition of miR-372 by *HULC* led to reduced translational repression of its target gene, *PRKACB*, which in turn induced phosphorylation of transcription factor CREB [102,160].

**Figure 2:**
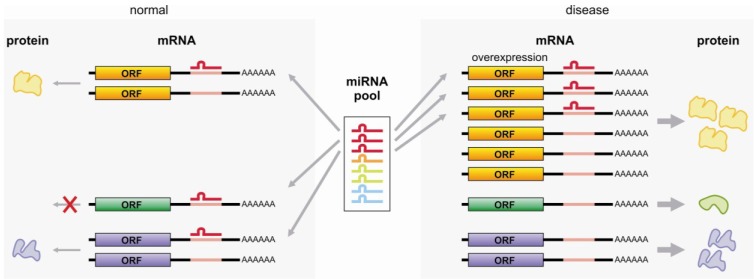
miRNA sponge function for mRNA. In a normal cell, a specific miRNA can target a number of mRNAs resulting in the inhibition of translation and/or degradation of these transcripts. When the expression levels of one of the mRNAs targeted by this miRNA is changed, a redistribution of the specific miRNA will cause a change in protein translation for multiple transcripts. In this schematic figure, the overexpressed yellow mRNA functions as a sponge for the red miRNA, yielding increased green and blue protein levels. In contrast, a depletion of the yellow miRNA sponge would result in a decrease in green, blue and yellow protein levels.

Similarly, two mRNA transcripts were recently shown to act as miRNA sponges: the 3' UTR regions of *Versican* (*VCAN*) mRNA in hepatocellular carcinoma (HCC) and of *CD44* mRNA in breast cancer cells [103,104]. The elevated levels of *VCAN* mRNA in HCC and HepG2 cells sequester miR-133a, miR-199a*, miR-144 and miR-431, thereby increasing the protein levels of amongst others CD34 and fibronectin (FN1), which have similar miRNA binding sites in their 3' UTRs [103]. Increased levels of the 3' UTR of *VCAN* increased proliferation, survival, migration, invasion, colony formation, and enhanced endothelial cell growth, but decreased apoptosis [103]. Similarly, *CD44* mRNA is elevated in breast cancer cells and its 3' UTR harbors binding sites for miR-328, miR-512-3p, miR-491 and miR671 [104]. Elevated *CD44* (3' UTR) levels sequester these miRNAs thereby increasing the protein levels of amongst others COL1α1 and fibronectin 1 (FN1), and enhanced the cell motility, invasion and cell adhesion and metastasis. Figure 2 shows a schematic representation of the miRNA sponge function of mRNA molecules. Importantly, by binding these miRNAs, the UTR sequences not only regulate their own transcript level homeostasis, they may also affect other transcripts by changing the available pool of these miRNAs through their decoy function [161]. Dynamics in this mode of regulation can be obtained by changing the length of the 3' UTR. For example, rapidly proliferating cells express shortened 3' UTRs, thereby decreasing the available positions for miRNA to bind [162].

### 3.5. RNA Degradation

Global transcriptome analyses has provided evidence that a large proportion of the genome can simultaneously produce transcripts from both strands, and that antisense transcripts commonly link “neighboring genes” in complex loci into chains of linked transcriptional units [163]. According to data generated by the FANTOM3 project, 4,520 pairs of full-length transcripts were able to form sense/antisense pairs on exons as detected in the mouse genome. Among them, 1,687 pairs were formed between protein coding genes, 2,478 by protein-coding/non-coding gene pairs and 355 by non-coding genes only [163]. Expression profiling revealed frequent concordant regulation of these sense/antisense pairs. One of the possible mechanisms for this transcript-mediated gene regulation is based on the sense-antisense RNA duplex formation. These sense-antisense transcript pairs can be regarded as Natural Antisense Transcripts (NATs). NATs are simply RNAs containing sequences that are complementary to other endogenous RNAs [105]. These can occur in *cis*, as described above, but they can also be transcribed in *trans* from separate loci (*trans*-NATs). Both *cis*- and *trans*-NATs can affect gene expression at the level of transcription, maturation, transport, stability and translation [105]. Numerous examples of *cis*- and *trans*-acting lncRNAs base-pairing with mRNA molecules and affecting its stability or translation have been describe so far [8,59,106,164,165,166].

A recently discovered group of *trans*-acting lncRNAs, termed *half-STAU1-binding site RNAs* (*½‑sbsRNAs*), can activate the decay of specific target mRNAs. Staufen 1 (STAU1)-mediated messenger RNA decay (SMD) involves the degradation of translationally active mRNAs upon STAU1 binding to the 3' UTR via double-stranded RNA [60]. STAU1-binding sites are formed by imperfect base-pairing between an Alu element in the 3' UTR of an mRNA target and an Alu element in a cytoplasmic lncRNA [60]. Evidently, Alu elements are highly needed to form RNA duplexes between mRNA and lncRNA that can be recognized by STAU1. As many mRNAs contain Alu elements in their 3' UTRs, it is highly plausible that also direct mRNA-mRNA base pairing may be a substrate for STAU1-mediated decay. A bioinformatic analysis revealed many stretches of imperfect base-pairing between Alu sequences localized within 5' and 3' UTR regions of mRNAs, similar to the *½-sbsRNAs* mode of action [105]. Whether such putative mRNA-mRNA pairings are functional and act via the SMD pathway will be the topic of future research.

### 3.6. Translational Control

LncRNAs are best known for their roles as regulators of transcription. However, recent studies have shown an important role of long non-coding RNAs in mRNA translation [8,106,108,164]. LncRNAs can modulate translation by two different mechanisms. As mentioned above, the *cis*- and *trans*-acting lncRNAs are capable to pair with mRNA molecules forming double-stranded RNA structures and thus inhibiting mRNA translation [8,106]. Alternatively, lncRNAs can act by affecting the general translation machinery [108]. *LincRNA-p21* is an example of a *trans*-acting lncRNA involved in translation inhibition [8,167]. The transcripts *CTNNB1* and *JUNB* (encoding β-catenin and JunB, respectively) base-pair imperfectly with *lincRNA-p21* at several places throughout the coding regions and UTRs. The formed *lincRNA-p21*-mRNA complex further interacts with translation repressors Rck and Fmrp, suggesting that *lincRNA-p21* can repress the translation of target mRNAs by operating via multiple mechanisms [8,167]. Another example of a *cis*-acting lncRNAs is antisense mRNA for *PU.1* gene [106,168]. The processed antisense RNA in the cytoplasm can bind to the sense *PU.1* transcript and stall translation between initiation and elongation steps [106,168]. 

Protein-coding antisense mRNA transcripts are also capable to form RNA duplexes with sense mRNA molecules leading to translation inhibition. *Antisense BCMA* RNA is transcribed from the same locus as *BCMA* and has typical mRNA features, e.g., polyadenylation, splicing, Kozak consensus sequence and an open reading frame encoding an experimentally proven 115 amino acid peptide: p12 protein [107]. Experimental data suggests that *antisense BCMA* inhibits the expression of BCMA protein, while it does not affect the expression level of *BCMA* mRNA. The inhibition of BCMA expression is obtained through the action of the antisense RNA and not of the p12 protein, although the exact mechanism is not fully understood [107]. 

A ncRNA that acts by affecting the general translation machinery is the *Xenopus laevis* transcript *BC1*. *BC1* transcript—expressed in neurons and germ cells—inhibits the assembly of the translation initiation complex [169]. The 3' region of the *BC1* RNA interacts with eIF4A and PABP and disrupts the functional link between the two factors which is necessary for efficient translation in *Xenopus* oocytes [108]. A near-complete restoration of translation occurs after introduction of excess eIF4A and PABP, indicating that translation repression by *BC1* happens via eIF4A and PABP [108].

The ability to inhibit general translation machinery is also identified for several mRNAs. These transcripts mainly act through the interaction of their UTRs with the RNA-dependent protein kinase (PKR). PKR is a serine-threonine protein kinase that is activated by intermolecular autophosphorylation upon binding to RNA molecules. The 3' UTR regions of cytoskeletal muscle mRNAs can act as *trans-*regulators by inhibiting translation through the activation of PKR [109]. Specifically, the 3' UTRs of tropomyosin, troponin and cardiac actin mRNAs can induce muscle cell differentiation and appear to function as tumor suppressors. These RNA sequences are predicted to form secondary structures with extended duplex stretches. It was shown that the 3' UTRs of cytoskeletal mRNAs interact with the RNA-binding domain of the PKR [109]. Once activated, PKR phosphorylates its substrates, including translation initiation factor eIF2α, which results in sequestration of another initiation factor, eIF2β, ultimately leading to inhibition of protein synthesis [109]. An important observation from this study is that full-length mRNA transcripts are more efficient at inhibiting translation than only their 3' UTR regions, suggesting the entire transcript is required for proper functioning [109]. Similarly, the *P23/TCTP* full-length mRNA but not a truncated version thereof, was able to bind and activate PKR, resulting in the inhibition of translation [110]. Several other protein coding transcripts have been reported to interact with PKR through their structured UTRs: the 5' UTRs of *VEGFA* mRNA [111] and *IFN-γ* mRNA [112], and the 3′ UTRs of *TPM1* mRNA [113] and *TNF-α* mRNA [114]. In all cases PKR activation caused inhibition of translation, which can have a *cis* effect on the translation level of mRNA itself as well as more general *trans* effect on the translation level of other transcripts.

Another mRNA with translational control is the tumor suppressor gene *p53* [115]. This gene is mutated in about half of all cancers and therefore considered a driver mutation gene [170,171]. The p53 protein works mainly as a transcription factor that acts upon cellular stresses such as DNA damage, stress of the endoplasmic reticulum (ER), hypoxia and telomere erosion [172]. When p53 is induced by this cellular stress, it can *trans*-activate a variety of target genes which promote cell cycle arrest, senescence or apoptosis [173,174]. Another p53 target with a different function is the *MDM2* gene. Its protein product is an E3 ubiquitin ligase which promotes polyubiquitination and proteasomal degradation of p53, thereby forming a negative regulatory feedback loop [175,176,177]. Interestingly, MDM2 is also involved in a positive regulatory feedback loop of p53. The mRNA of *p53* can interact with the RING domain of MDM2, which prevents the E3 ligase activity and furthermore stimulates translation of the *p53* mRNA [115]. At first, the interaction between the MDM2 protein and *p53* mRNA was considered to control the function of MDM2 [115]. Later it was demonstrated that phosphorylation of the Ser395 residue of MDM2 is required for the *p53* mRNA-MDM2 interaction and thereby acts as the switch for MDM2 between being a negative or a positive regulator [178].

### 3.7. Unknown Function

Recently, an example of a regulatory lncRNA in prostate cancer was described, with a proven functionally, but through a yet unknown mechanism of action [116]. In this high throughput RNA-sequencing study on clinical prostate cancer samples, a panel of 121 transcriptionally deregulated lncRNAs (*Prostate Cancer-Associated Transcripts*, or *PCATs*) were identified, representing potentially functional lncRNAs associated with prostate cancer. One of these transcripts, called *PCAT-1* was selectively upregulated only in prostate cancer and shown to function predominantly as a transcriptional repressor by facilitating *trans*-regulation of genes preferentially involved in mitosis and cell division, including known tumor suppressor genes, such as *BRCA2* [116].

Also several mRNAs, and more specifically their UTRs, have been reported to function as regulators (riboregulators) that suppress tumor formation but through unknown mechanisms. Results from Rastinejad and Blau suggest that the 3' UTRs of certain differentiation-specific RNAs are *trans*-acting regulators in feedback loops that inhibit cell division and promote differentiation [179]. More recently, the 3' UTR of several other transcripts were shown to reduce proliferation and induce differentiation of both myogenic cells and fibroblasts. The 3' UTR of *prohibitin* (*PHB*), an inhibitor of cell proliferation, significantly suppresses the tumorigenic properties and metastatic phenotype of transformed MCF7 cells [117]. Similarly, the 3' UTR of *ribonucleotide reductase* (*RNR*), a key rate-limiting enzyme in DNA synthesis, significantly suppresses the tumorigenic properties and metastatic phenotype of transformed fibroblasts cells [118]. Also the 5' UTR can fulfill such actions: the 5' UTR of the human *c-myc* P0 transcript suppresses the malignant phenotype of human breast cancer cells with decreased anchorage-independent proliferation, enhanced susceptibility to programmed cell death, and complete loss of the ability to form tumors in the intact animal [119]. For all these cases mentioned above, it is clear the UTRs harbor *trans*-regulatory functions, but the exact mechanism of their action is currently still unknown. 

## 4. Epigenetic Regulatory Potential of Protein-Coding RNA

It is well known that many lncRNAs are involved in the regulation of gene expression at the epigenetic level. Approximately 20–30% of all lncRNAs have been shown to be able to physically interact with specific epigenetic enzymes, which control the reversible modification of histone residues and DNA methylation, thereby influencing the activity of genes [120,180]. Upon binding, the lncRNAs can guide chromatin modifying complexes to their target regions. Such lncRNAs can guide either gene activators (for example the lncRNAs *HOTTIP* [64] or *Mistral* [181]) or gene repressors (e.g., *HOTAIR* [55], *HOTAIRM1* [120], *ANRIL* [15,57], *Kcnq1ot1* [56], *Air* [121], *Xist* [182] or *pRNA* [61]). LncRNAs can even function as a scaffold, bringing together multiple protein partners to form ribonucleoprotein complexes, which are subsequently guided to their genomic target locations. For example, *HOTAIR* can simultaneously bind to both the polycomb repressive complex 2 (PRC2) and the LSD1-CoREST complex using specific domains of the RNA molecule [55], while *ANRIL* and *HOTAIRM1* directly interact with proteins from both PRC1 and PRC2 complexes [15,57,120]. Similarly, the lncRNA *Kcnq1ot1* interacts with both the PRC2 and G9a (EHMT2) to lay down the silencing histone marks H3K27me3 and H3K9me2, respectively [56]. In recent attempts to characterize all RNA molecules that interact with the PRC2 complex, RNA immunoprecipitation experiments combined with next generation sequencing have been conducted by us and others [29,122,183]. Thus far, these studies have mainly focused on the interactions between lncRNAs and PRC2 complex components. Zhao and colleagues focused mainly on imprinted non-coding transcripts and *MEG3* in particular, which directs PRC2 to the reciprocally imprinted *Dlk1* coding gene [122]. Guil *et al*. only describe results for non-coding intronic RNA sequences [29]. They report several intronic RNA regions capable of interacting with PRC2 components and inducing repression of the host gene *in cis*. One of their examples is the *SMYD3* intronic RNA, which can bind to EZH2, a component of the PRC2 complex, thereby targeting this repressive complex to the *SMYD3* gene. SMYD3 is a SET domain-containing H3K4 methyltransferase with oncogenic properties, which is frequently overexpressed in colorectal, breast and liver cancer [184,185]. Reducing the levels of SMYD3 by *SMYD3* intronic RNA, resulted in reduced tumor growth, and revealed *SMYD3* intronic RNA to harbor tumor suppressive abilities [29]. Similarly, several other intronic RNAs with stand-alone regulatory functions were recently described in mice, implicating this to be a common type of multi-functionality within mammalian (primary) transcripts [186]. Finally, in experiments from our own laboratory, we analyzed the binding ability of transcripts over 200 nucleotides in size to SUZ12, one of the PRC2 complex components, in prostate cancer cells [183]. Both SUZ12 and EZH2 proteins are part of the PRC2 complex, contain RNA binding domains and have been shown to interact with RNA molecules [55,57,182,187].

**Figure 3:**
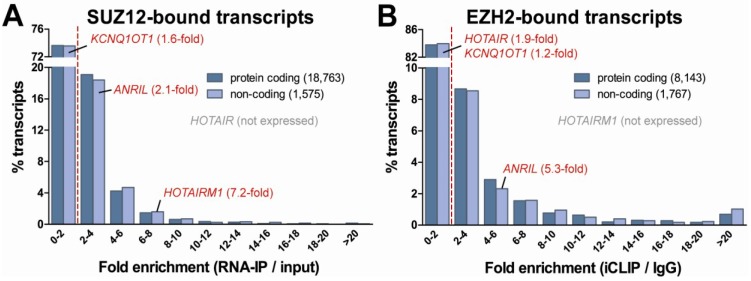
RNA binding to PRC2 complex components. (**A**) Analysis of data from our lab showed that both mRNAs and lncRNAs bind to the PRC2 complex component SUZ12 with similar binding affinities [183]. For comparison, known lncRNA-PRC2 interactions and their fold enrichments are shown in red. Here, the RNA-IP experiments were performed on the prostate cancer cell line LNCaP upon formaldehyde-fixation; (**B**) Reanalysis of the raw data from the Guil *et al*. confirmed our finding that both protein-coding and non-coding RNA can bind with high affinity to the PRC2 complex, in this case the EZH2 subunit [29]. These data were obtained from UV cross-linking experiments (iCLIP) in the colorectal cancer cell line HCT116.

To specifically gain insight into the binding of protein-coding RNA molecules to the PRC2 complex, we initially compared results for both mRNAs and lncRNAs in experiments from our own laboratory. In these experiments, we determined the SUZ12-bound RNA fraction in the human prostate cancer cell line LNCaP upon formaldehyde-fixation (RNA-IP) via next-generation sequencing and compared these results to input material [183]. To our surprise, protein-coding transcripts appeared to bind to the PRC2 complex with similar affinities as lncRNAs did. In fact, a substantial portion of mRNAs (and lncRNAs) bound with even stronger affinities to PRC2 than previously reported lncRNA-PRC2 interactions, including *HOTAIRM1*, *ANRIL* and *KCNQ1OT1* (Figure 3A). Independent replicates reproduced our initial findings. Next, we decided to reanalyze the raw data from similar experiments from the Esteller laboratory [29]. In these experiments EZH2-RNA interactions were studied in the human colorectal cancer cell line HCT116 by UV cross-linking (iCLIP) and next-generation sequencing. We compared the levels of EZH2-bound transcripts with background levels (IgG-bound fraction) to calculate fold-enrichment values. This reanalysis confirmed the findings from our own experimental data, and showed similar enrichment levels for mRNAs and lncRNAs, again with many transcripts binding stronger than known lncRNA interactors (Figure 3B). The (re-)analysis of data from both the Esteller lab and our lab yielded very similar results, even though both studies were conducted in different cancer cell lines, targeting different PRC2 complex components and using different experimental set ups. Finally, we included results from the Zhao *et al*. study, in which mouse embryonic stem cells were used to identify RNAs that interacted with the PRC2 complex component EZH2 via immunoprecipitation and next-generation sequencing [122]. There, over 9,000 transcripts were detected that interacted with EZH2, including many protein-coding genes (Table 2). Even though the depth of sequencing in this study was much lower than the study by Guil *et al.* and our study, their data also showed frequent enrichments of protein-coding transcripts, in particular those encoding for oncogenes and tumor suppressors, similar to transcripts from imprinted genes.

**Table 2:** EZH2-binding transcripts in mouse ES cells. Table is adapted from Zhao and colleagues [122].

Gene type	% enriched	# enriched	# total examined
**lncRNAs**	**10.2%**	216	2,127
**Oncogenes**	**44.3%**	182	411
**Tumor Suppresor Genes**	**41.0%**	325	793
**Imprinted Genes**	**41.0%**	34	83

**Figure 4:**
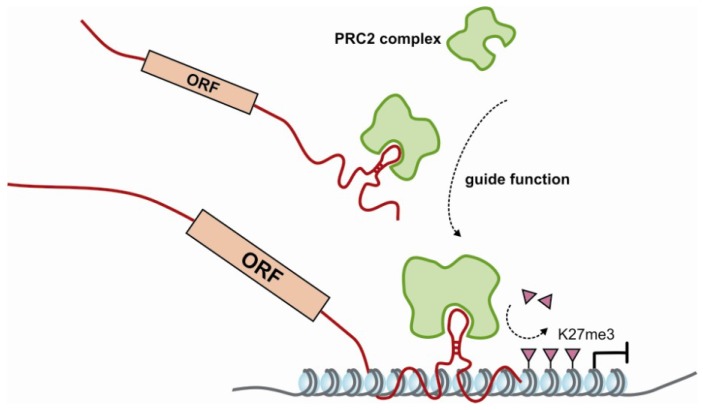
Proposed guide function for mRNA. Many mRNAs have here been shown to interact with PRC2 complex components. Similar to lncRNAs, we propose that mRNAs are involved in guiding the PRC2 complex to its target locations in the genome, where it can repress genomic regions by depositing a trimethyl mark on the lysine 27 residue of histone H3 (K27me3). Which part of the mRNA directly interacts with the PRC2 complex is currently not known.

In conclusion, all three studies described above imply a vast level of interaction between proteins of the PRC2 complex and protein-coding RNAs. These results are also in line with recent mRNA-proteome interaction studies where mRNAs appear to interact with regulatory enzymes and proteins. In these large proteome studies hundreds of mRNA binding proteins were identified [188,189]. As expected, the list of RNA binders was enriched for already known RNA binding proteins, involved in mRNA splicing, localization, processing and translation. However, also proteins functioning in transcription regulation were clearly identified, including transcription factors and co-activators (such as MYBBP1A and EDF) [188]. What functions these RNA-protein interactions have and by what mechanism these proteins may modulate transcription remains to be determined. Here, we hypothesize that mRNAs such as those binding to the PRC2 complex can indeed have additional regulatory functions (Figure 4). Currently, we cannot rule out the possibility that these mRNA-PRC2 interactions are non-specific events, but their levels of enrichment in all three studies are similar to or even stronger than known functional lncRNA-PRC2 interactions. Further studies are needed to prove a functional role for these mRNA-PRC2 interactions.

## 5. Conclusions

From the vast amount of papers it is clear that long non-coding RNA can have a variety of important roles in gene deregulation in cancer. Evidence of similar roles for protein-coding transcripts is now slowly accumulating. Here, we have combined, reviewed and extended the current knowledge of *trans*-regulatory roles for mRNA. Side-by-side, we have compared lncRNA and mRNA examples with similar regulatory functions. We have shown that mRNAs can frequently be associated with the PRC2 complex components and hypothesize a common guiding role for mRNA molecules. Future experiments need to further substantiate these speculations. Lastly, conclusions from loss-of-function experiments for mRNAs may need to be reinterpreted as the effects may not automatically be solely attributed to the associated protein function, but instead may also be partially due to affected regulatory functions. Again, further experimentation will show the extent of these regulatory roles for coding RNA.

## References

[1] Gilbert W. (1986). Origin of life—The RNA world. Nature.

[2] Joyce G.F. (2002). The antiquity of RNA-based evolution. Nature.

[3] Orgel L.E. (2004). Prebiotic chemistry and the origin of the RNA world. Crit. Rev. Biochem. Mol. Biol..

[4] Berg J.M., Tymoczko J.L., Stryer L. (2003). Biochemistry.

[5] The_ENCODE_Project_Consortium (2012). An integrated encyclopedia of DNA elements in the human genome. Nature.

[6] Harrow J., Frankish A., Gonzalez J.M., Tapanari E., Diekhans M., Kokocinski F., Aken B.L., Barrell D., Zadissa A., Searle S. (2012). Gencode: The reference human genome annotation for the encode project. Genome Res..

[7] Huarte M., Guttman M., Feldser D., Garber M., Koziol M.J., Kenzelmann-Broz D., Khalil A.M., Zuk O., Amit I., Rabani M. (2010). A large intergenic noncoding RNA induced by p53 mediates global gene repression in the p53 response. Cell.

[8] Yoon J.H., Abdelmohsen K., Srikantan S., Yang X.L., Martindale J.L., De S., Huarte M., Zhan M., Becker K.G., Gorospe M. (2012). Lincrna-p21 suppresses target mRNA translation. Mol. Cell.

[9] Huarte M., Rinn J.L. (2010). Large non-coding RNAs: Missing links in cancer?. Hum. Mol. Genet..

[10] Gutschner T., Diederichs S. (2012). The hallmarks of cancer a long non-coding RNA point of view. RNA Biol..

[11] Gupta R.A., Shah N., Wang K.C., Kim J., Horlings H.M., Wong D.J., Tsai M.C., Hung T., Argani P., Rinn J.L. (2010). Long non-coding RNA hotair reprograms chromatin state to promote cancer metastasis. Nature.

[12] Ji P., Diederichs S., Wang W., Boing S., Metzger R., Schneider P.M., Tidow N., Brandt B., Buerger H., Bulk E. (2003). Malat-1, a novel noncoding RNA, and thymosin beta4 predict metastasis and survival in early-stage non-small cell lung cancer. Oncogene.

[13] Mourtada-Maarabouni M., Pickard M.R., Hedge V.L., Farzaneh F., Williams G.T. (2009). Gas5, a non-protein-coding RNA, controls apoptosis and is downregulated in breast cancer. Oncogene.

[14] Zhang X., Gejman R., Mahta A., Zhong Y., Rice K.A., Zhou Y., Cheunsuchon P., Louis D.N., Klibanski A. (2010). Maternally expressed gene 3, an imprinted noncoding RNA gene, is associated with meningioma pathogenesis and progression. Cancer Res..

[15] Yap K.L., Li S.D., Munoz-Cabello A.M., Raguz S., Zeng L., Mujtaba S., Gil J., Walsh M.J., Zhou M.M. (2010). Molecular interplay of the noncoding RNA *ANRIL* and methylated histone H3 lysine 27 by polycomb CBX7 in transcriptional silencing of *INK4a*. Mol. Cell.

[16] Yu W.Q., Gius D., Onyango P., Muldoon-Jacobs K., Karp J., Feinberg A.P., Cui H.M. (2008). Epigenetic silencing of tumour suppressor gene p15 by its antisense RNA. Nature.

[17] Gibb E.A., Vucic E.A., Enfield K.S., Stewart G.L., Lonergan K.M., Kennett J.Y., Becker-Santos D.D., MacAulay C.E., Lam S., Brown C.J. (2011). Human cancer long non-coding rna transcriptomes. PLoS One.

[18] Calin G.A., Croce C.M. (2006). MicroRNA signatures in human cancers. Nat. Rev. Cancer.

[19] Esquela-Kerscher A., Slack F.J. (2006). Oncomirs—MicroRNAs with a role in cancer. Nat. Rev. Cancer.

[20] Croce C.M. (2009). Causes and consequences of microRNA dysregulation in cancer. Nat. Rev. Genet..

[21] Esteller M. (2011). Non-coding rnas in human disease. Nat. Rev. Genet..

[22] Mattick J.S., Makunin I.V. (2005). Small regulatory rnas in mammals. Hum. Mol. Genet..

[23] Guttman M., Amit I., Garber M., French C., Lin M.F., Feldser D., Huarte M., Zuk O., Carey B.W., Cassady J.P. (2009). Chromatin signature reveals over a thousand highly conserved large non-coding RNAs in mammals. Nature.

[24] Derrien T., Johnson R., Bussotti G., Tanzer A., Djebali S., Tilgner H., Guernec G., Martin D., Merkel A., Knowles D.G. (2012). The gencode v7 catalog of human long noncoding RNAs: Analysis of their gene structure, evolution, and expression. Genome Res..

[25] Kapranov P., Cheng J., Dike S., Nix D.A., Duttagupta R., Willingham A.T., Stadler P.F., Hertel J., Hackermuller J., Hofacker I.L. (2007). RNA maps reveal new RNA classes and a possible function for pervasive transcription. Science.

[26] Dinger M.E., Pang K.C., Mercer T.R., Mattick J.S. (2008). Differentiating protein-coding and noncoding RNA: Challenges and ambiguities. PLoS Comput. Biol..

[27] Sana J., Faltejskova P., Svoboda M., Slaby O. (2012). Novel classes of non-coding RNAs and cancer. J. Transl. Med..

[28] Dieci G., Fiorino G., Castelnuovo M., Teichmann M., Pagano A. (2007). The expanding RNA polymerase III transcriptome. Trends Genet..

[29] Guil S., Soler M., Portela A., Carrere J., Fonalleras E., Gomez A., Villanueva A., Esteller M. (2012). Intronic RNAs mediate EZH2 regulation of epigenetic targets. Nat. Struct. Mol. Biol..

[30] Furuno M., Pang K.C., Ninomiya N., Fukuda S., Frith M.C., Bult C., Kai C., Kawai J., Carninci P., Hayashizaki Y. (2006). Clusters of internally primed transcripts reveal novel long noncoding RNAs. PLoS Genet..

[31] Djebali S., Davis C.A., Merkel A., Dobin A., Lassmann T., Mortazavi A., Tanzer A., Lagarde J., Lin W., Schlesinger F. (2012). Landscape of transcription in human cells. Nature.

[32] Lin M.F., Jungreis I., Kellis M. (2011). PhyloCSF: A comparative genomics method to distinguish protein coding and non-coding regions. Bioinformatics.

[33] Bánfai B., Jia H., Khatun J., Wood E., Risk B., Gundling W.E., Kundaje A., Gunawardena H.P., Yu Y., Xie L. (2012). Long noncoding RNAs are rarely translated in two human cell lines. Genome Res..

[34] Okazaki Y., Furuno M., Kasukawa T., Adachi J., Bono H., Kondo S., Nikaido I., Osato N., Saito R., Suzuki H. (2002). Analysis of the mouse transcriptome based on functional annotation of 60,770 full-length cDNAs. Nature.

[35] Imanishi T., Itoh T., Suzuki Y., O'Donovan C., Fukuchi S., Koyanagi K.O., Barrero R.A., Tamura T., Yamaguchi-Kabata Y., Tanino M. (2004). Integrative annotation of 21,037 human genes validated by full-length cDNA clones. PLoS Biol..

[36] Dinger M.E., Gascoigne D.K., Mattick J.S. (2011). The evolution of RNAs with multiple functions. Biochimie.

[37] Prasanth K.V., Spector D.L. (2007). Eukaryotic regulatory RNAs: An answer to the “genome complexity” conundrum. Genes Dev..

[38] Frith M.C., Forrest A.R., Nourbakhsh E., Pang K.C., Kai C., Kawai J., Carninci P., Hayashizaki Y., Bailey T.L., Grimmond S.M. (2006). The abundance of short proteins in the mammalian proteome. PLoS Genet..

[39] Odermatt A., Taschner P.E., Scherer S.W., Beatty B., Khanna V.K., Cornblath D.R., Chaudhry V., Yee W.C., Schrank B., Karpati G. (1997). Characterization of the gene encoding human sarcolipin (SLN), a proteolipid associated with serca1: Absence of structural mutations in five patients with brody disease. Genomics.

[40] Klaudiny J., von der Kammer H., Scheit K.H. (1992). Characterization by cdna cloning of the mRNA of a highly basic human protein homologous to the yeast ribosomal protein yl41. Biochem. Biophys. Res. Commun..

[41] Galindo M.I., Pueyo J.I., Fouix S., Bishop S.A., Couso J.P. (2007). Peptides encoded by short ORFs control development and define a new eukaryotic gene family. PLoS Biol..

[42] Clamp M., Fry B., Kamal M., Xie X.H., Cuff J., Lin M.F., Kellis M., Lindblad-Toh K., Lander E.S. (2007). Distinguishing protein-coding and noncoding genes in the human genome. Proc. Natl. Acad. Sci. USA.

[43] Lindblad-Toh K., Garber M., Zuk O., Lin M.F., Parker B.J., Washietl S., Kheradpour P., Ernst J., Jordan G., Mauceli E. (2011). A high-resolution map of human evolutionary constraint using 29 mammals. Nature.

[44] Duret L., Chureau C., Samain S., Weissenbach J., Avner P. (2006). The xist RNA gene evolved in eutherians by pseudogenization of a protein-coding gene. Science.

[45] Brosch M., Saunders G.I., Frankish A., Collins M.O., Yu L., Wright J., Verstraten R., Adams D.J., Harrow J., Choudhary J.S. (2011). Shotgun proteomics aids discovery of novel protein-coding genes, alternative splicing, and “resurrected” pseudogenes in the mouse genome. Genome Res..

[46] Niazi F., Valadkhan S. (2012). Computational analysis of functional long noncoding rnas reveals lack of peptide-coding capacity and parallels with 3' UTRs. RNA.

[47] Wan Y., Qu K., Ouyang Z., Kertesz M., Li J., Tibshirani R., Makino D.L., Nutter R.C., Segal E., Chang H.Y. (2012). Genome-wide measurement of RNA folding energies. Mol. Cell.

[48] Hannon G.J., Rivas F.V., Murchison E.P., Steitz J.A. (2006). The expanding universe of noncoding RNAs. Cold Spring Harb. Symp. Quant. Biol..

[49] Mercer T.R., Dinger M.E., Mattick J.S. (2009). Long non-coding RNAs: Insights into functions. Nat. Rev. Genet..

[50] Ponting C.P., Oliver P.L., Reik W. (2009). Evolution and functions of long noncoding rnas. Cell.

[51] Mercer T.R., Mattick J.S. (2013). Structure and function of long noncoding RNAs in epigenetic regulation. Nat. Struct. Mol. Biol..

[52] Qiu M.T., Hu J.W., Yin R., Xu L. (2013). Long noncoding RNA: An emerging paradigm of cancer research. Tumour Biol..

[53] Martianov I., Ramadass A., Serra Barros A., Chow N., Akoulitchev A. (2007). Repression of the human dihydrofolate reductase gene by a non-coding interfering transcript. Nature.

[54] Kino T., Hurt D.E., Ichijo T., Nader N., Chrousos G.P. (2010). Noncoding RNA gas5 is a growth arrest- and starvation-associated repressor of the glucocorticoid receptor. Sci. Signal..

[55] Tsai M.C., Manor O., Wan Y., Mosammaparast N., Wang J.K., Lan F., Shi Y., Segal E., Chang H.Y. (2010). Long noncoding RNA as modular scaffold of histone modification complexes. Science.

[56] Pandey R.R., Mondal T., Mohammad F., Enroth S., Redrup L., Komorowski J., Nagano T., Mancini-Dinardo D., Kanduri C. (2008). *Kcnq1ot1* antisense noncoding RNA mediates lineage-specific transcriptional silencing through chromatin-level regulation. Mol. Cell.

[57] Kotake Y., Nakagawa T., Kitagawa K., Suzuki S., Liu N., Kitagawa M., Xiong Y. (2011). Long non-coding RNA anril is required for the PRC2 recruitment to and silencing of p15(INK4B) tumor suppressor gene. Oncogene.

[58] Guttman M., Rinn J.L. (2012). Modular regulatory principles of large non-coding RNAs. Nature.

[59] Faghihi M.A., Modarresi F., Khalil A.M., Wood D.E., Sahagan B.G., Morgan T.E., Finch C.E., St. Laurent G., Kenny P.J., Wahlestedt C. (2008). Expression of a noncoding RNA is elevated in alzheimer’s disease and drives rapid feed-forward regulation of beta-secretase. Nat. Med..

[60] Gong C., Maquat L.E. (2011). LncRNAs transactivate STAU1-mediated mrna decay by duplexing with 3' UTRs via Alu elements. Nature.

[61] Schmitz K.M., Mayer C., Postepska A., Grummt I. (2010). Interaction of noncoding RNA with the rDNA promoter mediates recruitment of DNMT3B and silencing of rRNA genes. Genes Dev..

[62] Aguilera A., Garcia-Muse T. (2012). R loops: From transcription byproducts to threats to genome stability. Mol. Cell.

[63] Jeon Y., Lee J.T. (2011). YY1 tethers Xist RNA to the inactive X nucleation center. Cell.

[64] Wang K.C., Yang Y.W., Liu B., Sanyal A., Corces-Zimmerman R., Chen Y., Lajoie B.R., Protacio A., Flynn R.A., Gupta R.A. (2011). A long noncoding RNA maintains active chromatin to coordinate homeotic gene expression. Nature.

[65] Rinn J.L., Chang H.Y. (2012). Genome regulation by long noncoding RNAs. Annu. Rev. Biochem..

[66] Wang K.C., Chang H.Y. (2011). Molecular mechanisms of long noncoding RNAs. Mol. Cell.

[67] Baldassarre A., Masotti A. (2012). Long non-coding RNAs and p53 regulation. Int. J. Mol. Sci..

[68] Da Sacco L., Baldassarre A., Masotti A. (2012). Bioinformatics tools and novel challenges in long non-coding RNAs (lncRNAs) functional analysis. Int. J. Mol. Sci..

[69] Chen G., Wang Z., Wang D., Qiu C., Liu M., Chen X., Zhang Q., Yan G., Cui Q. (2013). Lncrnadisease: A database for long-non-coding Rna-associated diseases. Nucleic Acids Res..

[70] Chen J.Z., Yang T., Yu H., Sun K., Shi Y., Song W.H., Bai Y.Y., Wang X.J., Lou K.J., Song Y. (2010). A functional variant in the 3'-UTR of angiopoietin-1 might reduce stroke risk by interfering with the binding efficiency of microRNA 211. Hum. Mol. Genet..

[71] Delay C., Calon F., Mathews P., Hebert S.S. (2011). Alzheimer-specific variants in the 3' UTR of amyloid precursor protein affect microrna function. Mol. Neurodegener..

[72] Wilkie G.S., Dickson K.S., Gray N.K. (2003). Regulation of mrna translation by 5'- and 3'-UTR-binding factors. Trends Biochem. Sci..

[73] Kochetov A.V., Ischenko I.V., Vorobiev D.G., Kel A.E., Babenko V.N., Kisselev L.L., Kolchanov N.A. (1998). Eukaryotic mrnas encoding abundant and scarce proteins are statistically dissimilar in many structural features. FEBS Lett..

[74] Pickering B.M., Willis A.E. (2005). The implications of structured 5' untranslated regions on translation and disease. Semin. Cell Dev. Biol..

[75] Eulalio A., Huntzinger E., Izaurralde E. (2008). Getting to the root of miRNA-mediated gene silencing. Cell.

[76] Lee I., Ajay S.S., Yook J.I., Kim H.S., Hong S.H., Kim N.H., Dhanasekaran S.M., Chinnaiyan A.M., Athey B.D. (2009). New class of microrna targets containing simultaneous 5'-UTR and 3'-UTR interaction sites. Genome Res..

[77] Lytle J.R., Yario T.A., Steitz J.A. (2007). Target mRNAs are repressed as efficiently by microRNA-binding sites in the 5' UTR as in the 3' UTR. Proc. Natl. Acad. Sci. USA.

[78] Zhang L.N., Liu Y.X., Song F.J., Zheng H., Hu L.M., Lu H., Liu P.F., Hao X.S., Zhang W., Chen K.X. (2011). Functional SNP in the microrna-367 binding site in the 3' UTR of the calcium channel ryanodine receptor gene 3 (RYR3) affects breast cancer risk and calcification. Proc. Natl. Acad. Sci. USA.

[79] Valgardsdottir R., Chiodi F., Giordano M., Cobianchi F., Riva S., Biamonti G. (2005). Structural and functional characterization of noncoding repetitive RNAs transcribed in stressed human cells. Mol. Biol. Cell.

[80] Clemson C.M., Hutchinson J.N., Sara S.A., Ensminger A.W., Fox A.H., Chess A., Lawrence J.B. (2009). An architectural role for a nuclear noncoding RNA: Neat1 RNA is essential for the structure of paraspeckles. Mol. Cell.

[81] Shevtsov S.P., Dundr M. (2011). Nucleation of nuclear bodies by RNA. Nat. Cell Biol..

[82] Kloc M., Wilk K., Vargas D., Shirato Y., Bilinski S., Etkin L.D. (2005). Potential structural role of non-coding and coding RNAs in the organization of the cytoskeleton at the vegetal cortex of xenopus oocytes. Development.

[83] Zhou Y., Zhong Y., Wang Y., Zhang X., Batista D.L., Gejman R., Ansell P.J., Zhao J., Weng C., Klibanski A. (2007). Activation of p53 by MEG3 non-coding RNA. J. Biol. Chem..

[84] Tripathi V., Ellis J.D., Shen Z., Song D.Y., Pan Q., Watt A.T., Freier S.M., Bennett C.F., Sharma A., Bubulya P.A. (2010). The nuclear-retained noncoding RNA MALAT1 regulates alternative splicing by modulating SR splicing factor phosphorylation. Mol. Cell.

[85] Colley S.M., Leedman P.J. (2009). Sra and its binding partners: An expanding role for RNA-binding coregulators in nuclear receptor-mediated gene regulation. Crit. Rev. Biochem. Mol. Biol..

[86] Lanz R.B., McKenna N.J., Onate S.A., Albrecht U., Wong J.M., Tsai S.Y., Tsai M.J., O’Malley B.W. (1999). A steroid receptor coactivator, SRA, functions as an RNA and is present in an SRC-1 complex. Cell.

[87] Deblois G., Giguere V. (2003). Ligand-independent coactivation of er alpha AF-1 by steroid receptor RNA activator (SRA) via MAPK activation. J. Steroid Biochem. Mol. Biol..

[88] Zhao X.S., Patton J.R., Davis S.L., Florence B., Ames S.J., Spanjaard R.A. (2004). Regulation of nuclear receptor activity by a pseudouridine synthase through posttranscriptional modification of steroid receptor rna activator. Mol. Cell.

[89] Hatchell E.C., Colley S.M., Beveridge D.J., Epis M.R., Stuart L.M., Giles K.M., Redfern A.D., Miles L.E.C., Barker A., MacDonald L.M. (2006). SLIRP, a small SRA binding protein, is a nuclear receptor corepressor. Mol. Cell.

[90] Caretti G., Schiltz R.L., Dilworth F.J., Di Padova M., Zhao P., Ogryzko V., Fuller-Pace F.V., Hoffman E.P., Tapscott S.J., Sartorelli V. (2006). The RNA helicases p68/p72 and the noncoding RNA SRA are coregulators of MyoD and skeletal muscle differentiation. Dev. Cell.

[91] Hube F., Velasco G., Rollin J., Furling D., Francastel C. (2011). Steroid receptor rna activator protein binds to and counteracts SRA RNA-mediated activation of MyoD and muscle differentiation. Nucleic Acids Res..

[92] Watanabe M., Yanagisawa J., Kitagawa H., Takeyama K., Ogawa S., Arao Y., Suzawa M., Kobayashi Y., Yano T., Yoshikawa H. (2001). A subfamily of RNA-binding DEAD-box proteins acts as an estrogen receptor ALPHA coactivator through the *N*-terminal activation domain (AF-1) with an RNA coactivator, SRA. EMBO J..

[93] Zhao X.S., Patton J.R., Ghosh S.K., Fischel-Ghodsian N., Shen L., Spanjaard R.A. (2007). Pus3p-and Pus1p-dependent pseudouridylation of steroid receptor RNA activator controls a functional switch that regulates nuclear receptor signaling. Mol. Endocrinol..

[94] Lanz R.B., Razani B., Goldberg A.D., O'Malley B.W. (2002). Distinct RNA motifs are important for coactivation of steroid hormone receptors by steroid receptor RNA activator (SRA). Proc. Natl. Acad. Sci. USA.

[95] Yik J.H., Chen R., Nishimura R., Jennings J.L., Link A.J., Zhou Q. (2003). Inhibition of P-TEFb (CDK9/Cyclin T) kinase and RNA polymerase II transcription by the coordinated actions of HEXIM1 and 7SK snRNA. Mol. Cell.

[96] Egloff S., van Herreweghe E., Kiss T. (2006). Regulation of polymerase ii transcription by 7SK snRNA: Two distinct rna elements direct P-TEFb and HEXIM1 binding. Mol. Cell. Biol..

[97] Nguyen V.T., Kiss T., Michels A.A., Bensaude O. (2001). 7SK small nuclear rna binds to and inhibits the activity of CDK9/Cyclin T complexes. Nature.

[98] Barboric M., Kohoutek J., Price J.P., Blazek D., Price D.H., Peterlin B.M. (2005). Interplay between 7SK snRNA and oppositely charged regions in HEXIM1 direct the inhibition of P-Tefb. EMBO J..

[99] He N., Jahchan N.S., Hong E., Li Q., Bayfield M.A., Maraia R.J., Luo K., Zhou Q. (2008). A La-related protein modulates 7SK snRNP integrity to suppress P-TEFb-dependent transcriptional elongation and tumorigenesis. Mol. Cell.

[100] Young T.M., Tsai M., Tian B., Mathews M.B., Pe’ery T. (2007). Cellular mrna activates transcription elongation by displacing 7SK RNA. PLoS One.

[101] Poliseno L., Salmena L., Zhang J.W., Carver B., Haveman W.J., Pandolfi P.P. (2010). A coding-independent function of gene and pseudogene mRNAs regulates tumour biology. Nature.

[102] Wang J.Y., Liu X.F., Wu H.C., Ni P.H., Gu Z.D., Qiao Y.X., Chen N., Sun F.Y., Fan Q.S. (2010). CREB up-regulates long non-coding RNA, HULC expression through interaction with microRNA-372 in liver cancer. Nucleic Acids Res..

[103] Fang L., Du W.W., Yang X., Chen K., Ghanekar A., Levy G., Yang W., Yee A.J., Lu W.Y., Xuan J.W. (2013). Versican 3'-untranslated region (3'-UTR) functions as a ceRNA in inducing the development of hepatocellular carcinoma by regulating mirna activity. FASEB J..

[104] Rutnam Z.J., Yang B.B. (2012). The non-coding 3' UTR of CD44 induces metastasis by regulating extracellular matrix functions. J. Cell Sci..

[105] Wang P., Yin S., Zhang Z., Xin D., Hu L., Kong X., Hurst L.D. (2008). Evidence for common short natural trans sense-antisense pairing between transcripts from protein coding genes. Genome Biol..

[106] Ebralidze A.K., Guibal F.C., Steidl U., Zhang P., Lee S., Bartholdy B., Jorda M.A., Petkova V., Rosenbauer F., Huang G. (2008). Pu.1 expression is modulated by the balance of functional sense and antisense RNAs regulated by a shared cis-regulatory element. Genes Dev..

[107] Hatzoglou A., Deshayes F., Madry C., Lapree G., Castanas E., Tsapis A. (2002). Natural antisense RNA inhibits the expression of BCMA, a tumour necrosis factor receptor homologue. BMC Mol. Biol..

[108] Wang H., Iacoangeli A., Lin D., Williams K., Denman R.B., Hellen C.U., Tiedge H. (2005). Dendritic BC1 RNA in translational control mechanisms. J. Cell Biol..

[109] Nussbaum J.M., Gunnery S., Mathews M.B. (2002). The 3'-untranslated regions of cytoskeletal muscle mrnas inhibit translation by activating the double-stranded rna-dependent protein kinase PKR. Nucleic Acids Res..

[110] Bommer U.A., Borovjagin A.V., Greagg M.A., Jeffrey I.W., Russell P., Laing K.G., Lee M., Clemens M.J. (2002). The mRNA of the translationally controlled tumor protein p23/TCTP is a highly structured RNA, which activates the dsRNA-dependent protein kinase PKR. RNA.

[111] Masuda K., Teshima-Kondo S., Mukaijo M., Yamagishi N., Nishikawa Y., Nishida K., Kawai T., Rokutan K. (2008). A novel tumor-promoting function residing in the 5' non-coding region of vascular endothelial growth factor mRNA. PLoS Med..

[112] Ben-Asouli Y., Banai Y., Pel-Or Y., Shir A., Kaempfer R. (2002). Human interferon-gamma mrna autoregulates its translation through a pseudoknot that activates the interferon-inducible protein kinase PKR. Cell.

[113] Davis S., Watson J.C. (1996). *In vitro* activation of the interferon-induced, double-stranded RNA-dependent protein kinase PKR by RNA from the 3' untranslated regions of human alpha-tropomyosin. Proc. Natl. Acad. Sci. USA.

[114] Osman F., Jarrous N., Ben-Asouli Y., Kaempfer R. (1999). A *cis*-acting element in the 3'-untranslated region of human TNF-alpha mRNA renders splicing dependent on the activation of protein kinase PKR. Genes Dev..

[115] Candeias M.M., Malbert-Colas L., Powell D.J., Daskalogianni C., Maslon M.M., Naski N., Bourougaa K., Calvo F., Fahraeus R. (2008). P53 mRNA controls p53 activity by managing Mdm2 functions. Nat. Cell Biol..

[116] Prensner J.R., Iyer M.K., Balbin O.A., Dhanasekaran S.M., Cao Q., Brenner J.C., Laxman B., Asangani I.A., Grasso C.S., Kominsky H.D. (2011). Transcriptome sequencing across a prostate cancer cohort identifies PCAT-1, an unannotated lincRNA implicated in disease progression. Nat. Biotechnol..

[117] Manjeshwar S., Branam D.E., Lerner M.R., Brackett D.J., Jupe E.R. (2003). Tumor suppression by the prohibitin gene 3' untranslated region RNA in human breast cancer. Cancer Res..

[118] Fan H., Villegas C., Huang A., Wright J.A. (1996). Suppression of malignancy by the 3' untranslated regions of ribonucleotide reductase R1 and R2 messenger RNAs. Cancer Res..

[119] Blume S.W., Miller D.M., Guarcello V., Shrestha K., Meng Z., Snyder R.C., Grizzle W.E., Ruppert J.M., Gartland G.L., Stockard C.R. (2003). Inhibition of tumorigenicity by the 5'-untranslated RNA of the human *c-myc* P0 transcript. Exp. Cell Res..

[120] Guttman M., Donaghey J., Carey B.W., Garber M., Grenier J.K., Munson G., Young G., Lucas A.B., Ach R., Bruhn L. (2011). Lincrnas act in the circuitry controlling pluripotency and differentiation. Nature.

[121] Nagano T., Mitchell J.A., Sanz L.A., Pauler F.M., Ferguson-Smith A.C., Feil R., Fraser P. (2008). The air noncoding RNA epigenetically silences transcription by targeting G9a to chromatin. Science.

[122] Zhao J., Ohsumi T.K., Kung J.T., Ogawa Y., Grau D.J., Sarma K., Song J.J., Kingston R.E., Borowsky M., Lee J.T. (2010). Genome-wide identification of polycomb-associated RNAs by RIP-seq. Mol. Cell.

[123] Alastalo T.P., Hellesuo M., Sandqvist A., Hietakangas V., Kallio M., Sistonen L. (2003). Formation of nuclear stress granules involves HSF2 and coincides with the nucleolar localization of Hsp70. J. Cell Sci..

[124] Biamonti G., Caceres J.F. (2009). Cellular stress and RNA splicing. Trends Biochem. Sci..

[125] Fox A.H., Lam Y.W., Leung A.K.L., Lyon C.E., Andersen J., Mann M., Lamond A.I. (2002). Paraspeckles: A novel nuclear domain. Curr. Biol..

[126] Hutchinson J.N., Ensminger A.W., Clemson C.M., Lynch C.R., Lawrence J.B., Chess A. (2007). A screen for nuclear transcripts identifies two linked noncoding RNAs associated with SC35 splicing domains. BMC Genomics.

[127] Sasaki Y.T.F., Ideue T., Sano M., Mituyama T., Hirose T. (2009). Men epsilon/beta noncoding rnas are essential for structural integrity of nuclear paraspeckles. Proc. Natl. Acad. Sci. USA.

[128] Marzluff W.F., Wagner E.J., Duronio R.J. (2008). Metabolism and regulation of canonical histone mRNAs: Life without a poly(a) tail. Nat. Rev. Genet..

[129] Cioce M., Lamond A.I. (2005). Cajal bodies: A long history of discovery. Annual Review of Cell and Developmental Biology.

[130] Matera A.G., Izaguire-Sierra M., Praveen K., Rajendra T.K. (2009). Nuclear bodies: Random aggregates of sticky proteins or crucibles of macromolecular assembly?. Dev. Cell.

[131] Heasman J., Wessely O., Langland R., Craig E.J., Kessler D.S. (2001). Vegetal localization of maternal mRNAs is disrupted by vegt depletion. Dev. Biol..

[132] Braconi C., Kogure T., Valeri N., Huang N., Nuovo G., Costinean S., Negrini M., Miotto E., Croce C.M., Patel T. (2011). MicroRNA-29 can regulate expression of the long non-coding RNA gene MEG3 in hepatocellular cancer. Oncogene.

[133] Benetatos L., Hatzimichael E., Dasoula A., Dranitsaris G., Tsiara S., Syrrou M., Georgiou I., Bourantas K.L. (2010). CPG methylation analysis of the MEG3 and snRPN imprinted genes in acute myeloid leukemia and myelodysplastic syndromes. Leuk. Res..

[134] Yamada K., Kano J., Tsunoda H., Yoshikawa H., Okubo C., Ishiyama T., Noguchi M. (2006). Phenotypic characterization of endometrial stromal sarcoma of the uterus. Cancer Sci..

[135] Lin R., Maeda S., Liu C., Karin M., Edgington T.S. (2007). A large noncoding RNA is a marker for murine hepatocellular carcinomas and a spectrum of human carcinomas. Oncogene.

[136] Tano K., Mizuno R., Okada T., Rakwal R., Shibato J., Masuo Y., Ijiri K., Akimitsu N. (2010). Malat-1 enhances cell motility of lung adenocarcinoma cells by influencing the expression of motility-related genes. FEBS Lett..

[137] Kawashima H., Takano H., Sugita S., Takahara Y., Sugimura K., Nakatani T. (2003). A novel steroid receptor co-activator protein (SRAP) as an alternative form of steroid receptor RNA-activator gene: Expression in prostate cancer cells and enhancement of androgen receptor activity. Biochem. J..

[138] Charette M., Gray M.W. (2000). Pseudouridine in RNA: What, where, how, and why. IUBMB Life.

[139] Shi Y.H., Downes M., Xie W., Kao H.Y., Ordentlich P., Tsai C.C., Hon M., Evans R.M. (2001). Sharp, an inducible cofactor that integrates nuclear receptor repression and activation. Genes Dev..

[140] Emberley E., Huang G.J., Hamedani M.K., Czosnek A., Ali D., Grolla A., Lu B., Watson P.H., Murphy L.C., Leygue E. (2003). Identification of new human coding steroid receptor RNA activator isoforms. Biochem. Biophys. Res. Commun..

[141] Hussein-Fikret S., Fuller P.J. (2005). Expression of nuclear receptor coregulators in ovarian stromal and epithelial tumours. Mol. Cell. Endocrinol..

[142] Lanz R.B., Chua S.S., Barron N., Soder B.M., DeMayo F., O’Malley B.W. (2003). Steroid receptor RNA activator stimulates proliferation as well as apoptosis *in vivo*. Mol. Cell. Biol..

[143] Leygue E., Dotzlaw H., Watson P.H., Murphy L.C. (1999). Expression of the steroid receptor RNA activator in human breast tumors. Cancer Res..

[144] Hube F., Guo J.M., Chooniedass-Kothari S., Cooper C., Hamedani M.K., Dibrov A.A., Blanchard A.A.A., Wang X.M., Deng G., Myal Y. (2006). Alternative splicing of the first intron of the steroid receptor RNA activator (SRA) participates in the generation of coding and noncoding RNA isoforms in breast cancer cell lines. DNA Cell Biol..

[145] Cooper C., Guo J.M., Yan Y., Chooniedass-Kothari S., Hube F., Hamedani M.K., Murphy L.C., Myal Y., Leygue E. (2009). Increasing the relative expression of endogenous non-coding steroid receptor RNA activator (SRA) in human breast cancer cells using modified oligonucleotides. Nucleic Acids Res..

[146] Murphy L.C., Simon S.L.R., Parkes A., Leygue E., Dotzlaw H., Snell L., Troup S., Adeyinka A., Watson P.H. (2000). Altered expression of estrogen receptor coregulators during human breast tumorigenesis. Cancer Res..

[147] Chooniedass-Kothari S., Hamedani M.K., Troup S., Hube F., Leygue E. (2006). The steroid receptor RNA activator protein is expressed in breast tumor tissues. Int. J. Cancer.

[148] Faust T., Frankel A., D’Orso I. (2012). Transcription control by long non-coding RNAs. Transcription.

[149] Wassarman D.A., Steitz J.A. (1991). Structural analyses of the 7SK ribonucleoprotein (RNP), the most abundant human small RNP of unknown function. Mol. Cell. Biol..

[150] Marz M., Donath A., Verstraete N., Nguyen V.T., Stadler P.F., Bensaude O. (2009). Evolution of 7SK RNA and its protein partners in metazoa. Mol. Biol. Evol..

[151] Krueger B.J., Jeronimo C., Roy B.B., Bouchard A., Barrandon C., Byers S.A., Searcey C.E., Cooper J.J., Bensaude O., Cohen E.A. (2008). Larp7 is a stable component of the 7SK snRNP while P-Tefb, hexim1 and hnRNP A1 are reversibly associated. Nucleic Acids Res..

[152] Markert A., Grimm M., Martinez J., Wiesner J., Meyerhans A., Meyuhas O., Sickmann A., Fischer U. (2008). The La-related protein LARP7 is a component of the 7SK ribonucleoprotein and affects transcription of cellular and viral polymerase II genes. EMBO Rep..

[153] Baek D., Villen J., Shin C., Camargo F.D., Gygi S.P., Bartel D.P. (2008). The impact of micrornas on protein output. Nature.

[154] Bartel D.P. (2009). Micrornas: Target recognition and regulatory functions. Cell.

[155] Ventura A., Jacks T. (2009). MicroRNAs and cancer: Short RNAs go a long way. Cell.

[156] Lujambio A., Lowe S.W. (2012). The microcosmos of cancer. Nature.

[157] Ebert M.S., Sharp P.A. (2010). Emerging roles for natural microRNA sponges. Curr. Biol..

[158] Franco-Zorrilla J.M., Valli A., Todesco M., Mateos I., Puga M.I., Rubio-Somoza I., Leyva A., Weigel D., Garcia J.A., Paz-Ares J. (2007). Target mimicry provides a new mechanism for regulation of microRNA activity. Nat. Genet..

[159] Cazalla D., Yario T., Steitz J.A. (2010). Down-regulation of a host microRNA by a herpesvirus saimiri noncoding RNA. Science.

[160] Panzitt K., Tschernatsch M.M., Guelly C., Moustafa T., Stradner M., Strohmaier H.M., Buck C.R., Denk H., Schroeder R., Trauner M. (2007). Characterization of HULC, a novel gene with striking up-regulation in hepatocellular carcinoma, as noncoding RNA. Gastroenterology.

[161] Almeida M.I., Reis R.M., Calin G.A. (2012). Decoy activity through microRNAs: The therapeutic implications. Expert Opin. Biol. Ther..

[162] Sandberg R., Neilson J.R., Sarma A., Sharp P.A., Burge C.B. (2008). Proliferating cells express mrnas with shortened 3' untranslated regions and fewer microRNA target sites. Science.

[163] Katayama S., Tomaru Y., Kasukawa T., Waki K., Nakanishi M., Nakamura M., Nishida H., Yap C.C., Suzuki M., Kawai J. (2005). Antisense transcription in the mammalian transcriptome. Science.

[164] Carrieri C., Cimatti L., Biagioli M., Beugnet A., Zucchelli S., Fedele S., Pesce E., Ferrer I., Collavin L., Santoro C. (2012). Long non-coding antisense RNA controls Uchl1 translation through an embedded SINEB2 repeat. Nature.

[165] Matsui K., Nishizawa M., Ozaki T., Kimura T., Hashimoto I., Yamada M., Kaibori M., Kamiyama Y., Ito S., Okumura T. (2008). Natural antisense transcript stabilizes inducible nitric oxide synthase messenger RNA in rat hepatocytes. Hepatology.

[166] Yanagida S., Taniue K., Sugimasa H., Nasu E., Takeda Y., Kobayashi M., Yamamoto T., Okamoto A., Akiyama T. (2013). ASBEL, an ANA/BTG3 antisense transcript required for tumorigenicity of ovarian carcinoma. Sci. Rep..

[167] Yoon J.H., Abdelmohsen K., Gorospe M. (2012). Posttranscriptional gene regulation by long noncoding RNA. J. Mol. Biol..

[168] Faghihi M.A., Wahlestedt C. (2009). Regulatory roles of natural antisense transcripts. Nat. Rev. Mol. Cell Biol..

[169] Kindler S., Wang H., Richter D., Tiedge H. (2005). RNA transport and local control of translation. Annu. Rev. Cell Dev. Biol..

[170] Hollstein M., Sidransky D., Vogelstein B., Harris C.C. (1991). P53 mutations in human cancers. Science.

[171] Soussi T., Wiman K.G. (2007). Shaping genetic alterations in human cancer: The p53 mutation paradigm. Cancer Cell.

[172] Levine A.J., Oren M. (2009). The first 30 years of p53: Growing ever more complex. Nat. Rev. Cancer.

[173] Vousden K.H., Prives C. (2009). Blinded by the light: The growing complexity of p53. Cell.

[174] Riley T., Sontag E., Chen P., Levine A. (2008). Transcriptional control of human p53-regulated genes. Nat. Rev. Mol. Cell Biol..

[175] Kubbutat M.H.G., Jones S.N., Vousden K.H. (1997). Regulation of p53 stability by MDM2. Nature.

[176] Haupt Y., Maya R., Kazaz A., Oren M. (1997). MDM2 promotes the rapid degradation of p53. Nature.

[177] Harris S.L., Levine A.J. (2005). The p53 pathway: Positive and negative feedback loops. Oncogene.

[178] Gajjar M., Candeias M.M., Malbert-Colas L., Mazars A., Fujita J., Olivares-Illana V., Fahraeus R. (2012). The p53 mRNA-MDM2 interaction controls MDM2 nuclear trafficking and is required for p53 activation following DNA damage. Cancer Cell.

[179] Rastinejad F., Blau H.M. (1993). Genetic complementation reveals a novel regulatory role for 3' untranslated regions in growth and differentiation. Cell.

[180] Khalil A.M., Guttman M., Huarte M., Garber M., Raj A., Morales D.R., Thomas K., Presser A., Bernstein B.E., van Oudenaarden A. (2009). Many human large intergenic noncoding rnas associate with chromatin-modifying complexes and affect gene expression. Proc. Natl. Acad. Sci. USA.

[181] Bertani S., Sauer S., Bolotin E., Sauer F. (2011). The noncoding RNA *mistral* activates *Hoxa6* and *Hoxa7* expression and stem cell differentiation by recruiting MLL1 to chromatin. Mol. Cell.

[182] Zhao J., Sun B.K., Erwin J.A., Song J.J., Lee J.T. (2008). Polycomb proteins targeted by a short repeat RNA to the mouse x chromosome. Science.

[183] Karapetyan A.R., Kuiper R.A., Coolen M.W. Department of Human Genetics, Nijmegen Centre for Molecular Life Sciences (NCMLS), Radboud University Nijmegen Medical Centre, P.O. Box 9101, Nijmegen 6500 HB, The Netherlands.

[184] Hamamoto R., Furukawa Y., Morita M., Iimura Y., Silva F.P., Li M., Yagyu R., Nakamura Y. (2004). SMYD3 encodes a histone methyltransferase involved in the proliferation of cancer cells. Nat. Cell Biol..

[185] Hamamoto R., Silva F.P., Tsuge M., Nishidate T., Katagiri T., Nakamura Y., Furukawa Y. (2006). Enhanced SMYD3 expression is essential for the growth of breast cancer cells. Cancer Sci..

[186] St. Laurent G., Shtokalo D., Tackett M.R., Yang Z., Eremina T., Wahlestedt C., Urcuqui-Inchima S., Seilheimer B., McCaffrey T.A., Kapranov P. (2012). Intronic RNAs constitute the major fraction of the non-coding RNA in mammalian cells. BMC Genomics.

[187] Kanhere A., Viiri K., Araujo C.C., Rasaiyaah J., Bouwman R.D., Whyte W.A., Pereira C.F., Brookes E., Walker K., Bell G.W. (2010). Short RNAs are transcribed from repressed polycomb target genes and interact with polycomb repressive complex-2. Mol. Cell.

[188] Baltz A.G., Munschauer M., Schwanhausser B., Vasile A., Murakawa Y., Schueler M., Youngs N., Penfold-Brown D., Drew K., Milek M. (2012). The mRNA-bound proteome and its global occupancy profile on protein-coding transcripts. Mol. Cell.

[189] Castello A., Fischer B., Eichelbaum K., Horos R., Beckmann B.M., Strein C., Davey N.E., Humphreys D.T., Preiss T., Steinmetz L.M. (2012). Insights into RNA biology from an atlas of mammalian mRNA-binding proteins. Cell.

